# Utilizing NMR fecal metabolomics as a novel technique for detecting the physiological effects of food shortages in waterfowl

**DOI:** 10.3389/fphys.2023.1229152

**Published:** 2024-01-10

**Authors:** Breanne A. Murray, Karen L. Machin

**Affiliations:** Department of Veterinary Biomedical Sciences, Western College of Veterinary Medicine, University of Saskatchewan, Saskatoon, SK, Canada

**Keywords:** stress physiology, waterfowl, metabolomics, fecal metabolomics, feed restriction, mallard duck, metabolism

## Abstract

Metabolomics is the study of small, endogenous metabolites that participate in metabolic reactions, including responses to stressors. Anthropogenic and environmental changes that alter habitat and food supply can act as stressors in wild waterfowl. These alterations invoke a series of physiological processes to provide energy to restore homeostasis and increase survival. In this study, we utilized fecal metabolomics to measure metabolites and identify pathways related to a 6-day feed restriction in captive mallard ducks (*Anas platyrhynchos*, n = 9). Fecal samples were collected before (baseline) and during feed restriction (treatment). H^1^ Nuclear Magnetic Resonance (NMR) spectroscopy was performed to identify metabolites. We found that fecal metabolite profiles could be used to distinguish between the feed-restricted and baseline samples. We identified metabolites related to pathways for energy production and metabolism endpoints, and metabolites indicative of gut microbiota changes. We also demonstrated that mallard ducks could utilize endogenous reserves in times of limited caloric intake. Fecal metabolomics shows promise as a non-invasive novel tool in identifying and characterizing physiological responses associated with stressors in a captive wild bird species.

## Introduction

The Prairie Pothole Region (PPR, a habitat composed of shallow ephemeral wetlands in 3 prairie provinces (Alberta, Saskatchewan and Manitoba) and five states) is one of North America’s most important habitats for nesting and migratory waterfowl (Mann, 1974). These wetlands are vulnerable and are experiencing drying due to climate change and are undergoing drainage for agricultural and urban development (Niemuth et al., 2014). Changes in this vital habitat are expected to cause waterfowl population declines through decreased nesting habitat and food availability (Niemuth et al., 2014).

Acquiring appropriate and sufficient nutritional resources is essential for wild populations during every life cycle stage (Fokidis et al., 2012). Shortages or unpredictable food availability is an environmental stressor for many wild species. Exposure to stressors causes the hypothalamic-pituitary-adrenal (HPA) axis to activate and release glucocorticoids (corticosterone (CORT) in birds) (Rich and Romero, 2005; Strochlic and Romero, 2008). When glucocorticoids are increased, they incite metabolic changes and biochemical responses, increasing available energy (accelerating the rate of gluconeogenesis) and improving the chance of survival (Teague et al., 2007). These metabolic alterations increase circulating glucose to help supply the increased energy demand and to overcome the stressor while temporarily suspending non-essential activities (e.g., digestion, growth) in favour of survival (MacDougall-Shackleton et al., 2019). Metabolic processes like gluconeogenesis, protein degradation, lipolysis, and metabolites such as glycerol, and ketone bodies are promoted to produce energy sources when circulating glucose levels are depleted (Teague et al., 2007).

While the short-lived physiological response to a stressor enables survival, chronic exposure to unpredictable or uncontrollable environmental challenges can result in prolonged CORT elevations that may have broad implications affecting survival (Sapolsky et al., 2000). With sustained chronic elevations of CORT, costly, non-essential processes (such as growth and reproduction) remain persistently inhibited in order to initiate pathways that produce energy to recover homeostasis (Wingfield et al., 1995; Blas, 2015). Consequently, the continued release of glucocorticoids reduces glycogen production and protein synthesis rates which can interfere with energy conservation through the persistence of catabolic actions, stimulating the degradation of fatty acids and proteins (Fokidis et al., 2012). Domestic chickens experiencing heat stress had a decrease in muscle protein synthesis and amino acid uptake, indicating muscle tissue catabolism and inhibition of growth (Lu et al., 2018). Another study in chickens exposed to heat stress found increased liver lipid metabolism, suggesting break down of fat stores for energy (Jastrebski et al., 2017). Similar studies in wild birds are lacking.

To better understand the metabolic consequences of the stress response, a process known as metabolomics can be utilized. Non-targeted metabolomics, a quantitative analysis of metabolites in biofluids (e.g., blood plasma or serum, urine, etc.), tissues or feces, can detect changes in an organism’s metabolome. The metabolome is a collection of metabolites that participate in metabolic reactions required for growth, maintenance, and responses to disease and stressors (Samuelsson and Larsson, 2008). Changes in the metabolome may reflect differences in organ function, thus giving vital clues about the physiological status and health of the individual (Viant, 2008). Over the past decade, there has been a rapid increase in environmental metabolomics studies to discover biomarkers of toxicant exposure and disease that can be applied to future conservation efforts (Viant et al., 2003). Nuclear magnetic resonance (NMR) spectrometry-based metabolomics is one of the dominant analytical methods used for environmental metabolomics studies The broad range of metabolites detected and ease of sample preparation makes NMR an easily reproducible tool making it suitable for repeated sampling for monitoring wildlife (Valerio et al., 2019).

Fecal metabolomics can not only provide quantification of the endpoints of metabolism but can also identify metabolites that result from the host-microbiome interaction and give an integrated picture of the metabolic consequences of the stress response. The gut microbiome, comprised of trillions of species of bacteria, can affect the host metabolism and energy homeostasis (Kogut, 2013; Schröder, 2022). Noguera et al. (2018), demonstrated that the composition of microbial community can be altered by glucocorticoids in a wild yellow-legged gulls (*Larus michahellis*) (Noguera et al., 2018). The metagenomic metabolite link between the gut and brain may be bidirectional through the gut-brain axis. In fact, gut microbiota are crucial for maintaining the homeostasis of the local, systemic, and brain systems (Bistoletti et al., 2020). The complex network between the HPA axis and autonomic nervous system facilitate communication between the gut and brain which regulates the host physiological homeostasis. Commensal gut bacteria-host interactions release endocrine messengers, neurotransmitters such as serotonin and gamma-aminobutyric acid (GABA), and metabolites such as short-chain fatty acids (SCFA) acetate, propionate, and butyrate which, in turn, influence brain function (Cryan et al., 2019).

To enhance our understanding of the physiological consequences of an ecologically relevant stressor, we subjected mallard ducks (*Anas platyrhynchos*) to 6 days of food restriction. This study aimed to quantify and identify fecal metabolites during chronic food shortages. We predicted that NMR spectrometry could be used to assess the fecal metabolome and distinguish fecal profiles of mallards before and during feed restriction. We hypothesize that ducks would utilize pathways to increase energy by metabolizing alternative sources such as fat and muscles during nutritional stress. Fecal metabolomics has rarely been applied in birds, although it has been used in mammals to non-invasively assess the consequences of stressors (Valerio et al., 2019). This is the first study to utilize fecal metabolomics in a wild waterfowl species and one of the first to be applied in a non-domestic species of birds.

## Materials and methods

To examine the metabolic response of waterfowl to a chronic stressor, a captive flock of adult (1 year old) mallard ducks (n = 9, 7 female, 2 male) were subjected to feed restriction for 6 days. At the start of the study (day 0), ducks had a mean weight of 1080 g ± 140 std dev (range 900–1350 g). Ducks were considered healthy based on a physical exam. No illnesses were noted during winter housing (October 2018-May 2019) or during the study. The ducks were housed in two outdoor fenced pens to mimic natural conditions 10 km south of St. Denis, Saskatchewan (52°06′32.5″N 106°04′25.7″W) in May 2019. The outdoor enclosure included upland grass (26.8 m^2^) and a natural pond (13.4 m^2^). These enclosures allowed ducks to exhibit natural nesting and dabbling behaviours. The pond area was lined, and pond water was pumped as needed to maintain adequate water levels and provide fresh water to the ducks. Access to pond water permitted supplemental feeding of natural aquatic invertebrates. Ducks were provided with a commercial nutritionally balanced diet providing 2850 kcal/kg, (crude protein (actual): 19%, crude fat (actual): 3.8%, crude fiber (actual): 5%) *ad libitum* except during food restriction. We followed the guidelines of the Canadian Council on Animal Care as defined by the Guide to the Care and Use of Experimental Animals. This project was approved by the University of Saskatchewan Animal Research Ethics Board—protocol no. 20030021.

**FIGURE 1:**
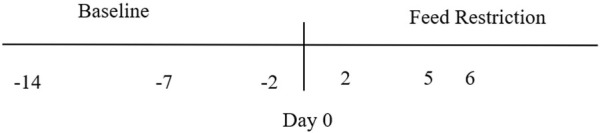
Sampling timeline for fecal collection from nine mallard ducks before (baseline) and during feed restriction. Baseline (19 samples), Day 2 (7 samples), Day 5 (7 samples), Day 6 (4 samples).

This study was divided into two activities: baseline (1) and food restriction (2) (Figure 1).

1) Fresh fecal samples were collected to characterize the baseline values of fecal metabolites 1 hour after the food was introduced in the morning on days −14, −7, −2. Baseline samples were taken over 2 weeks to ensure that repetitive handling did not impact the results and normal metabolome of the ducks was represented.
2) Ducks underwent a restricted food trial for 6 days to mimic a chronic stressful event where an uncontrolled environmental challenge leads to decreased food availability. Mallards were fed a diet that met 75% of their basal metabolic rate (BMR, minimum number of calories needed for the body to perform necessary functions) for 6 days. All ducks were weighed before (baseline days −14, −7, −2) and when the birds were sampled. The weight at the end of feed restriction was subtracted from the baseline weight (day 0) to calculate weight loss during the experiment.

BMR was calculated based on the weight of the birds during the baseline period using an equation developed for mallards (Prince, 1979). The BMR and metabolic energy of the feed were used to calculate the feed amount for all the birds daily. The mass of food (g) estimated to meet the BMR/bird/day was calculated.

$$\text{BMR }\left(\text{kcal}/\text{bird}/\text{day}\right)=87.9\;W^{0.734},\text{where }W=\text{mass }\left(\text{kg}\right)$$

The constant 87.9 (SE = 2.3) (Prince, 1979)

$$g={\text{BMR}}_{\text{bird}}/{\text{ME}}_{\text{Food}}$$

Feed was divided into individual bowls placed within a divided feeding structure to ensure that all ducks had equal access to food. Ducks were monitored for signs of aggression defined as chasing, pecking, feather pulling or fighting for a minimum of 20 min after introducing the feed. Fresh fecal samples were collected 1 h after the food was introduced in the morning during baseline and on days 2, 5 and 6 of the feed restriction period. We expected that fecal metabolite changes would not be immediate, so began sampling on day 2. As we were interested in chronic changes, we sampled again on days 5 and 6. We avoided daily sampling because we suspected frequent capture and handling would be stressful and potentially influence the results. Feces were collected by placing ducks into separate dry rectangular plastic storage boxes with plastic netting lids for airflow and ventilation on sampling days. Ducks were kept in fecal collection containers until they defecated but released from containers if they failed to defecate within 45 min of capture. Fecal samples were collected from the bottom of the fecal collection containers into cryovials and flash-frozen in liquid nitrogen to stop metabolite degradation. Samples were stored at −80 C until sample analysis. During feed restriction, ducks produced small and less frequent defecation which decreased the number of samples available for analysis.

Fecal samples were collected at baseline and on days 2, 5, and 6. Samples were collected at each time point with one or two birds a day exceeding the maximal allowable confinement time, preventing sufficient sample collection (Table 1). Each sample was lyophilized in a Labconco Freezone (Labcono Corporation, Kansas City, MO, United States) to remove water and standardize sample weight. However, day 6 samples were not analyzed as there was insufficient freeze-dried sample (minimum of 10 mg required) from the majority of the ducks (4/9 ducks). Freeze-dried fecal powder (10 mg) was extracted in random order to remove potential processing bias according to previously published protocols in chickens (Le Roy et al., 2016) using 500 µL of 0.2M pH7.4 + NaN_3_ 3 mM + trimethylsilylpropanoic acid - TMSP (C_6_H_14_O_2_Si Sigma Aldrich 269913) in 0.1% phosphate buffer in D2O. TMSP was used as a chemical shift reference as an internal standard. Then 200 µL of supernatant was pipetted into a 5 mm NMR stem tube for non-targeted ^1^H NMR spectroscopy (Bruker Advance III HD 600 MHz NMR spectrometer). Tuning and shimming was completed for each sample. A standard 1- dimensional noesypr1d pulse sequence (noesypr1d, 90° pulse length 15 µs, total acquisition time of 4 s) with water suppression applied during relaxation delay (1 s) and mixing time (100 m) at 298 k. For each sample, 128 scans were recorded.

**TABLE 1:** Sample collection and body mass changes for nince mallard ducks prior to (baseline) and after feed restriction.

Duck	Sex	Body mass day 0 (g)	Body mass post-feed restriction (g)	Body mass loss (g)	Percent body mass lost (%)	Number of samples collected during baseline	Sample collected on day 5 of feed restriction?
1	F	1200	756	444	37	3	Yes
2	F	1100	1000	100	9.1	3	No
3	F	950	900	50	5.3	1	Yes
4	M	1350	1200	150	11.1	3	Yes
5	F	1125	1000	125	11.1	3	Yes
6	M	1100	950	150	13.5	1	Yes
7	F	900	725	175	19.4	2	No
8	F	1050	850	200	19.0	1	Yes
9	F	950	850	100	10.5	2	Yes

To investigate variation in metabolite profiles among baseline and during feed restriction, raw NMR spectra were processed in Topspin 3.6 (Bruker Corporation, MA, United States) and then imported into Chenomx NMR Suite 7.0 (Chenomx, Edmonton, Canada) for manual baseline and shimming correction and TMSP was calibrated as 0.0 ppm. Peaks were assigned to individual metabolites by comparing chemical shifts of 1D 1H-NMR spectra of the samples to metabolites available at the Chenomx Compound Library. Metabolite profiling for samples was performed by a single person (B.A.M.) with the metabolite fitting algorithm available within the Chenomx software**.** Concentrations of compounds were exported into Excel and used for statistical analysis in MetaboAnalyst 5.0 (https://www.metaboanalyst.ca) (Pang et al., 2021).

Concentration data were normalized via constant sum; data were transformed with log normalization and mean-centered data scaling prior to statistical analysis. To ensure that repetitive handling did not impact the results we used a multiple-baseline design utilizing concentration data from every baseline day (Christ, 2007). There was no detectable difference in metabolome between days (ANOVA utilizing MetaboAnalyst identified 0 significant features) (baseline days −14, −7, −2) and will be referred to as baseline hereafter. Chemometric analyses of principal component analysis (PCA) and partial least squares discriminate analysis (PLS-DA) methods were applied to investigate differences in metabolite profiles between the baseline and food restriction periods. The PCA was used as an unsupervised approach to investigate the separation between samples, followed by a supervised PLS-DA to identify natural groupings and evaluate the difference between treatments. The PLS-DA model performance was validated using i) 10-fold CV, which used accuracy and R2 and Q2 values, and (ii) permutation tests, which used prediction accuracy after 100 iterations. When 10-fold CV values >0.6 and permutation test *p* < 0.05, PLS-DA models are considered accurate and predictive. The PLS-DA variable importance in projection plot (VIP) identified metabolites responsible for the variations between treatments. All metabolites with VIP score values greater than one were considered essential in separation between treatments. To determine if ducks with greater mass loss foraged more for alternative sources of food (i.e., insects (Becker et al., 1996)), we examined the relationship between trehalose and body mass loss using a Pearson’s product correlation.

The Pathway Analysis tool in MetaboAnlayst utilizing significant VIP metabolites (hereafter, VIP metabolites) was used to identify metabolic pathways. The human metabolome database (HMDB Version 4.0) was used to investigate the pathways associated with the identified metabolites. Pathway analysis was performed with MetaboAnalyst 5.0 using the chicken (*Gallus gallus*) database. Enrichment analysis was performed with over-representation analysis (ORA) was used to analyze the subset of metabolites identified with the VIP plot to identify the pathways associated with each sampling period (baseline vs feed restriction). The pathway library of the Kyoto Encyclopedia of genes and genomes (KEGG) database was used as the reference (Ogata et al., 1999).

## Results

Ducks did not exhibit any signs of aggression during feeding, before or during the food restriction period. During the 6-day feed restriction, ducks lost an average of 15% ± 9 of body mass (mean = 154.9 , SD = 63.9 range = 75 (6.9%) - 294 g (28%) Table 1). The Canadian Council on Animal Care (CCAC) considers 20% body mass reduction significant and in need of intervention. No duck reached this point prior to the last day of the study when they were provided with *ad-lib* food.

**FIGURE 2:**
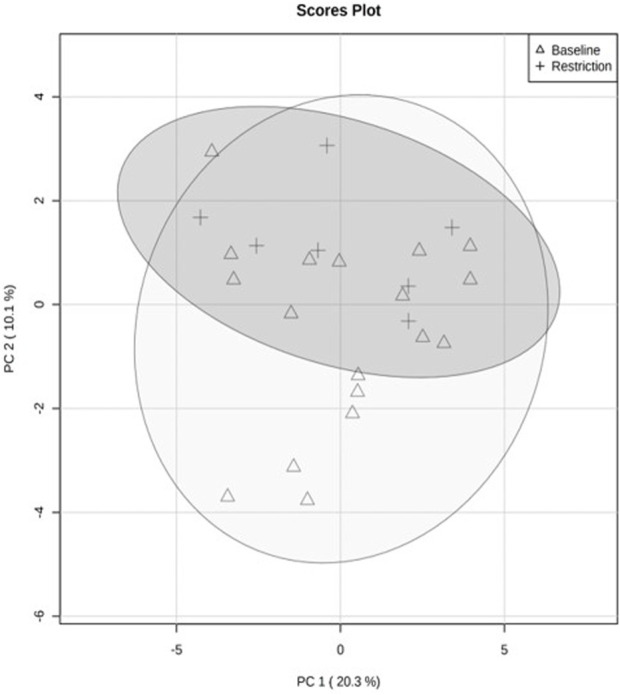
Quantitative analysis of fecal metabolite profiles from mallard ducks (n = 9) collected prior to (baseline days −14,-7,-2, 19 samples) and during feed restriction (day 5, 7 samples) using Principal Component Analysis (PCA). The separation of 95% confidence regions in clusters indicates a notable difference between metabolite profiles.

**FIGURE 3:**
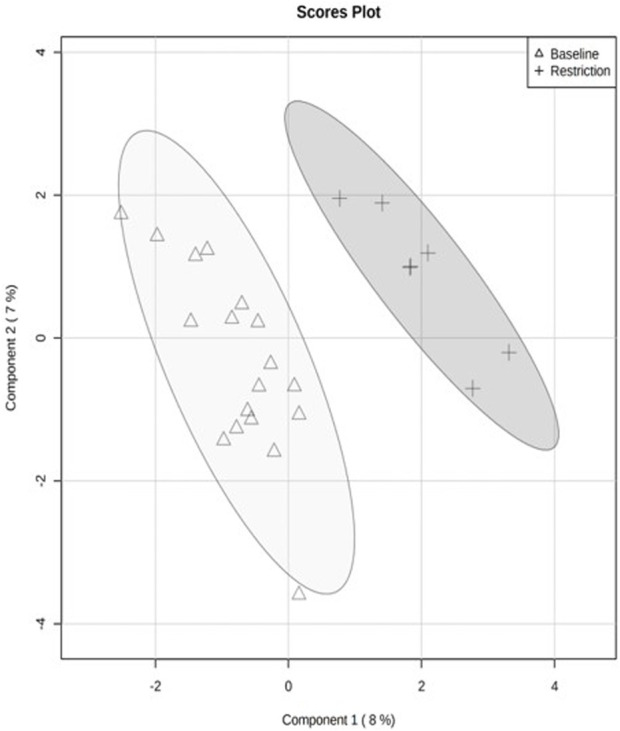
Quantitative analysis of fecal metabolite profiles from mallard ducks (n = 9) collected prior to (baseline days −14,-7,-2, 19 samples) and during feed restriction (day 5, 7 samples) using Partial Least Square Discriminant Analysis (PLS-DA). Separation of 95% confidence regions in clusters indicates a notable difference between metabolite profiles.

The PCA revealed separation between baseline and day 5 feed restriction metabolite profiles (Figure 2). The PCA fecal profiles started separating during the feed restriction day 2 (results not shown), and maximum separation was achieved on day 5. The two principal components made up 30.3% of the total variation (PC1 20.3% and PC2 10.1%). The PLS-DA showed separation between the baseline and feed restriction samples (Figure 3). The two principal components made up 15% of the total variation (PC1 8% and PC2 7%). The cross-validation of the PLS-DA model via leave-one-out cross-validation (LOOCV) showed that the first two components showed very high scores of accuracy (0.84). The multiple correlation coefficients (*R* ^2^, 0.91) and cross-validated *R* ^2^ (Q^2^, 0.17) indicated the PLS-DA model’s good fit and performance.

**FIGURE 4:**
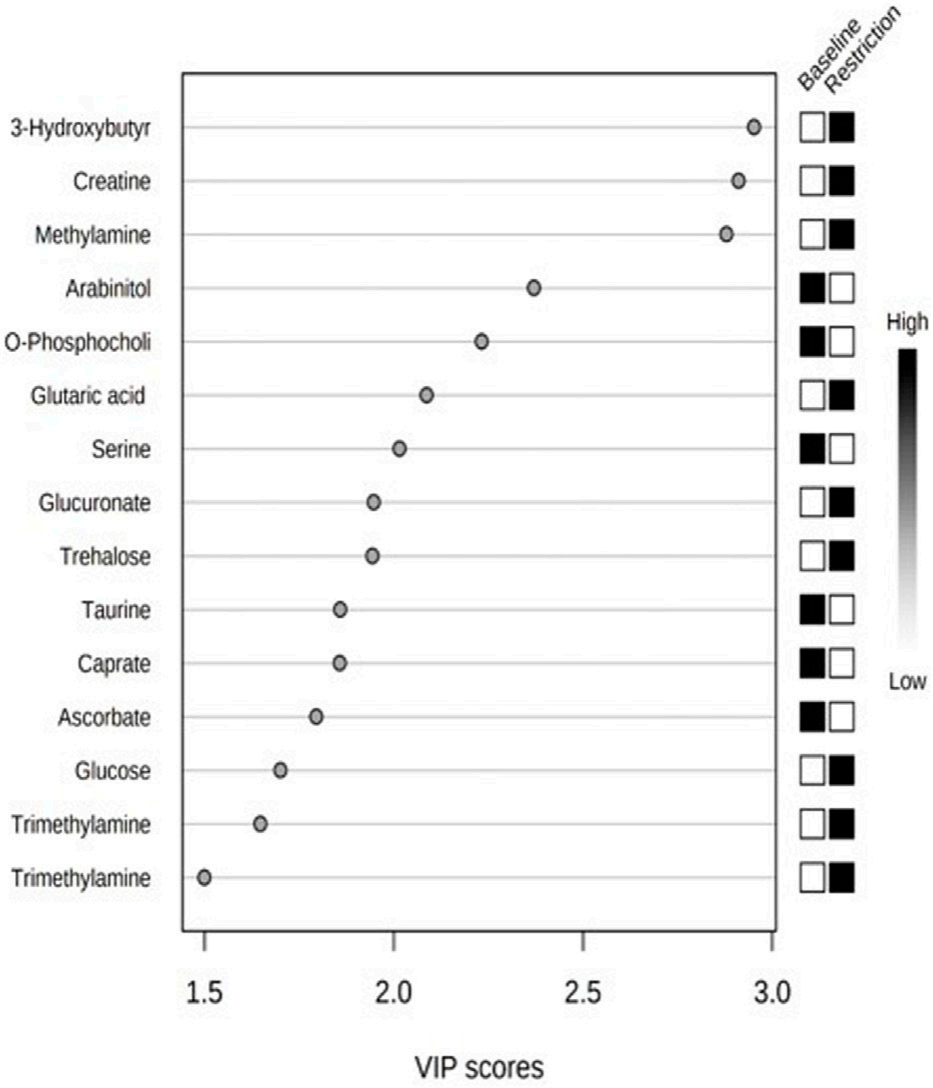
Top 14 metabolites based on variable importance in projection (VIP) scores from PLS-DA from mallard duck feces collected prior to (baseline days −14,-7,-2, 19 samples) and during feed restriction (day 5, 7 samples).

We putatively annotated 134 metabolites in the NMR spectra of the mallard feces. Variable importance in projection (VIP) scores from PLS-DA found 14 metabolites that explain the variation in metabolic profiles between treatments using the chicken (*Gallus gallus*) pathway library (Figure 4; Table 2). Compared to the baseline, mallard fecal sample during feed restriction contained higher concentrations of 3-hydroxybutyrate, creatine, methylamine, glucose, glutaric acid, trehalose, glucuronate, and trimethylamine and lower levels of arabinitol, O-Phosphocholine, ascorbate, caprate and amino acids (taurine, and serine) compared to baseline (Table 2). There was no relationship between trehalose and body mass loss (r = −0.331, *p* = 0.211).

**TABLE 2:** Top metabolites based on variable importance in projection (VIP) scores from PLS-DA from mallard duck feces collected prior to (baseline days −14, −7, −2, 19 samples) and during stress (food restriction day 5, 7 samples).

Metabolites decreased during feed restriction	Metabolites increased during feed restriction
•O-phosphocholine	•3-hydroxybutryrate
•Serine	•Creatine
•Taurine	•Methylamine
•Caparate	•Arabinitol
•Ascorbate	•Glutaric acid
	•Glucuronate
	•Trehalose
	•Glucose
	•Trimethylamine

We performed a metabolite site enrichment analysis (MSEA) with an over-representation analysis (ORA) to test if there were biologically relevant groups of metabolites. The MSEA is a method that helps to determine if metabolites identified in the VIP analysis are biologically relevant. This determination was made by identifying pathways or diseases associated with identified metabolites. The enrichment analysis uses prior knowledge of metabolites obtained through the Human Metabolome Database and identifies related pathways in the Kyoto Encyclopedia of Genes and Genomes (Xia et al., 2009). Metabolites with a higher concentration during the baseline period (arabinitol, O-phosphocholine, ascorbate, caprate, taurine, and serine) produced a metabolite set enrichment analysis that revealed the metabolites were associated with pathways related to taurine and hypotaurine metabolism, phosphatidylcholine biosynthesis, beta-oxidation of very-long-chain fatty acids, phospholipid biosynthesis, fatty acid biosynthesis, sphingolipid metabolism and bile acid biosynthesis. Metabolites with a higher concentration during the feed restriction period (3-hydroxybutyrate, creatine, methylamine, glutaric acid, glucose, trehalose, glucuronate, trimethylamine) produced a metabolite set enrichment analysis that revealed the metabolites were associated with pathways related to lactose degradation, trehalose degradation, glucose-alanine cycles, lactose synthesis, transfer of acetyl groups into mitochondria, glycolysis, starch and sucrose metabolism, inositol metabolism, gluconeogenesis, fatty acid biosynthesis, galactose metabolism, sphingolipid metabolism, arginine and proline metabolism, Warburg effect, glycine and serine metabolism and tyrosine metabolism.

## Discussion

In this study, we used NMR metabolomics to assess changes in mallard duck fecal metabolome exposed to an ecologically relevant stressor of chronic feed restriction. Using fecal metabolite profiles, we could differentiate between baseline and feed restriction, and 14 metabolites explained the variation between samples.

In order to maintain homeostasis, energy metabolism is required to provide continuous fuel for cellular processes (Wilson and Matschinsky, 2021). Carbohydrate metabolism is vital for energy production, the most essential being glucose (Scanes, 2022). Glucose is required for neuronal function (von Eugen et al., 2022). The brain is the most energy-intensive tissue, but avian neurons require approximately three times less glucose than their mammalian counterparts (von Eugen et al., 2022). Studies in birds have shown higher plasma glucose concentrations when compared to mammals that have similar body mass (Sweazea, 2022). Birds utilize glucose for various physiological functions such as cellular oxidation, glycogen (polysaccharide) synthesis (to store energy) in the liver and glycolytic flight muscles (pectoral and supracoracoideus), synthesis of fatty acids (lipogenesis) and other non-essential amino acids and vitamins (vitamin C) (Scanes, 2022).

Glucocorticoids promote hyperglycemia by activating multiple pathways to create energy. Glycerol is mobilized from tri- and di-glyceride lipids via lipolysis from adipose tissue, which can create glucose in the liver via gluconeogenesis. Glycogenolysis of glycogen in the skeletal muscles creates glucose-1-phosphate, which can be metabolized through glycolysis to maintain blood glucose levels (Goldstein et al., 1993; Landys et al., 2004). All mallards in our study exhibited a body mass reduction during feed restriction because energy expenditure was higher than metabolizable energy intake (75% of BMR). Although the mass loss between birds was variable, some individuals lost more than others despite receiving a similar feed allocation throughout the restriction period. This may have been due to individual genetics and invertebrate forging success among birds.

## Baseline

During the baseline period, metabolites identified were associated with phospholipid, bile acid, and fatty acid biosynthesis pathways and cell production. Amino acids, serine and taurine were higher during the baseline than during feed restriction. These metabolites form proteins and are essential for growth through the production of muscle and fat reserves (serine) and homeostatic maintenance through regulating digestion (taurine) (Kalhan and Hanson, 2012; Uyanga et al., 2022). Serine can be obtained from nutritional sources but can also be readily produced through the serine synthesis branch of glycolysis (Klaassen and Biebach, 1994). Ducks during feed restriction may have undergone serine deprivation, which has been shown to alter mitochondrial glucose and glutamine metabolism, reducing adenosine triphosphate (ATP) production (Gao et al., 2018). Declines in ATP can lead to a diminished growth rate, which may impede homeostasis. It is impossible to determine if depletion in serine occurred because of the inability to produce serine or if serine was utilized by the microbiota as an energy source when nutrients were limited (Velayudhan et al., 2004). More studies should be completed to investigate the turnover of serine in tissues during feed restriction. Taurine is an amino acid utilized in bile acid biosynthesis in the liver, facilitating the digestion of fats and oils. In chickens, taurine is typically regarded as a non-essential amino acid obtained through the diet and internal synthesis (Martin, 1972). However, when exposed to stressors, taurine may become semi-essential, meaning that the synthesis capacity may be insufficient to meet the increased demand. In these situations, taurine must be obtained through the diet. (Surai et al., 2020). Taurine increases glycogen synthesis, glycolysis and glucose uptake and inhibits gluconeogenic enzymes in the liver from releasing hepatic glucose (Batista et al., 2012).

Hepatic energy turnover is a dynamic process where the liver takes up, stores, and releases energy to maintain blood glucose concentrations to ensure energy supply to the tissues (Halperin and Cheema-Dhadli, 1989). As ducks were fed *ad lib* during baseline, substantial metabolism of hepatic energy reserves would not have occurred as there was sufficient glucose to maintain homeostasis. Interestingly, a human study found that plasma taurine increased after 3 days of fasting but decreased to baseline levels after 5 days of fasting (Tang et al., 2021). Those authors suggested that systemic metabolic reprogramming may occur after a period of continuous starvation (Tang et al., 2021). While the blood and fecal metabolomes are not the same, changes in the blood metabolome can be indicative of shifts or alterations in gut microbiota activity and various metabolic processes that may alter the fecal metabolome (Behr et al., 2019). Future work is needed in birds to determine if metabolic reprogramming occurs after a long fasting period. This flexibility may be a necessary physiological adaptation for migratory birds.

Multiple factors such as genetics, life-history stage, and environmental conditions can influence metabolic processes (Wingfield et al., 1998; Viant et al., 2003). Birds may rely on endogenous energy stores (e.g., glycogen, fatty acids, protein) during extended energetic effort and fasting (LaGrange and Dinsmore, 1988; Nord and Williams, 2015). Fat and muscle are essential energy reserves in long-distance migratory birds like mallards that experience prolonged periods of exertion where energetic costs are high. These life history stages are often accompanied by prolonged reductions in energy intake, limiting protein synthesis and leading to muscle catabolism and proteolysis (Moyano et al., 1998). Powered flight during migration causes the activation of flight muscles, which account for 10%–25% of the bird’s body mass, requiring large amounts of energy (DuBay et al., 2020). A study in garden warblers (*Sylvia borin*) found a 22% decrease in leg muscle mass after migration, likely through muscle catabolism needed to provide energy (Bauchinger and Biebach, 2005).

Higher concentrations of antioxidants associated with preventing cell damage, like ascorbate (ascorbic acid, vitamin C) and gut protectants, like O-phosphocholine, were observed during the baseline. Ascorbate is required for amino acid and mineral metabolism, which may be disrupted during periods of physiological stress (McDowell, 1989). A study in chickens exposed to heat stress demonstrated decreased ascorbate concentrations (Attia et al., 2016). Unlike mammals, birds can produce ascorbate in their liver, which is required to meet physiological needs through the enzymatic conversion of sugars (Khan et al., 2012). Under stressful conditions such as prolonged feed restriction, the ascorbate requirement may exceed endogenous production capacity, resulting in a net deficiency. During nutritional deprivation, birds may conserve endogenous sugars for neuronal function. O-phosphocholine involved in phospholipid biosynthesis, is found in cell membranes and is associated with a healthy gut (Wishart et al., 2018). Phosphatidylcholines are thought to protect the large intestine wall by producing a mucosal layer (Parlesak et al., 2007). This layer is a critical aspect of gut health and protects the intestines by creating a barrier against bacteria (Chen et al., 2015). Heat stress has been shown to cause gut barrier failure (Pearce et al., 2013). During feed restriction, possible gut mucosal barrier atrophy may have decreased gut health, although this was not assessed. A study in chickens found that gut segments had increased intestinal fragility (loss of tensile strength) with increasing duration of feed withdrawal, especially when longer than 14 h (Bilgili and Hess, 1997). Adequate tensile strength is crucial for gut health as increased fragility can lead to the leakage of digestive juices, bacteria, or undigested food into the abdominal cavity, potentially causing infection or inflammation (Egorov et al., 2002). Similarly a study in rats found short-term fasting-induced intestinal mucosal barrier atrophy, which may have occurred in feed-restricted ducks (Dock et al., 2004).

Medium-chain fatty acids have become increasingly popular in poultry feeds because of their antimicrobial effects (Çenesiz and Çiftci, 2020). Caprate (also called capric acid or decanoic acid) is a medium-chain fatty acid found in milk lipids and oils of several plants (Zentek et al., 2011). We observed a higher concentration of caprate during the baseline period. As caprate is a metabolite not synthesized by ducks, the presence of caprate in the feces is unknown but may have been present in the commercial diet. The caprate concentration in the feces was lower during feed restriction, likely due to reduced feed consumption.

## Feed restriction

Several extracellular and cellular mechanisms work together to form the mucosal barrier function to absorb nutrients. Many animals regulate glucose during feed restriction by mobilizing internal energy sources.

Mobilization of energy (glucose) can be achieved through the metabolizing of stored glycogen and alternative energy sources like amino acids (methylamine, 3-hydroxybutyrate, glucuronate, glutaric acid), through protein degradation and tissue metabolism (creatine), and bacterial utilization of alternative energy sources such as trehalose and trimethylamine (TMA). During fasting, metabolism will shift from homeostatic maintenance to increased utilization of energy reserves to increase glucose through glucocorticoid stimulation of gluconeogenesis in the liver (Sapolsky et al., 2000). During feed restriction; there was a significant increase in glucose in the feces. It is possible that a damaged mucosal barrier prevented the absorption of glucose. Jastrebski et al. (2017) found elevated glucose levels in the liver of birds exposed to chronic heat stress but did not measure fecal glucose (Jastrebski et al., 2017). In contrast, a study by Lu et al. (2018) found that birds exposed to chronic heat stress for 14 days exhibited lower serum glucose concentrations, possibly indicating exhaustion of the bird’s endogenous energy reserve (Lu et al., 2018). While ducks in this study were not heat stressed, environmental stressors are known to affect fecal metabolites in cattle (Valerio et al., 2019). This mobilization of energy reserves is beneficial in the short term, but long-term catabolism can lead to deleterious effects such as muscle wasting and death. In this experiment, methylamine was increased during feed restriction. Methylamine is an endogenous short aliphatic amine involved in the central regulation of food intake (Pirisino et al., 2004; Zendehdel et al., 2020)**.**

With food restriction and body mass loss, endogenous energy shortage requires mobilization of alternate energy sources to help maintain ATP synthesis like ketone bodies such as 3-hydroxybutyrate (β-Hydroxybutyric acid). Ketone bodies are produced in the liver when glucose from the glycerol reserve starts to decline, causing fatty acids and phospholipid metabolism (Mierziak et al., 2021). Thus, ketone increases may be beneficial in extended times of exertion, such as migration (Costantini, 2008). Numerous studies in migrant birds have reported increases in 3-hydroxybutyrate during flight (Landys et al., 2005; Bairlein et al., 2015; Gutiérrez et al., 2019). The amount of fuel stored before migration can limit migration distance (Gutiérrez et al., 2019). Mallards are capital breeders, meaning they arrive at the breeding grounds with the endogenous reserves needed for reproduction (Meijer and Drent, 1999). Thus, mallards will stop along migration flyways to replenish energy reserves depleted during flight (Beatty et al., 2017).

Incubation is another life history stage where female mallards rely on alternative energy sources, such as ketone bodies, as they lose an average of 25% of their body mass (Krapu, 1981). A study in nesting King eider (*Somateria spectabilis*) hens demonstrated reliance on endogenous and exogenous energy sources. Females had high concentrations of free fatty acids, β-hydroxybutyrate, and glycerol throughout incubation, indicating that fat reserves were a primary energy source (Bentzen et al., 2008). A study in king penguins (*Aptenodytes patagonicus*) demonstrated elevated 3-hydroxybutyrate concentrations after a 150-day natural fasting period, suggesting that ketone bodies are necessary to resist starvation (Cherel and Le Maho, 1985). While mallards are not as resistant to starvation, they appear to be able to utilize fat reserves to create energy during periods of food shortage.

In addition to ketone bodies, we observed higher concentrations of metabolites associated with pathways for alternative energy production, such as glucuronate (glucuronic acid), glutaric acid, and creatine, during feed restriction. This may demonstrate the mallard ducks’ ability to utilize endogenous reserves in times of limited caloric intake. Glucuronate is derived from glucose and is converted from less common sugars to ones that can be more readily metabolized to provide energy when ATP is limited (Bhagavan, 2002). Glucuronate is also a precursor of ascorbate (vitamin C). Glucuronate concentrations were higher during the feed restriction, while ascorbate was higher during the baseline period, indicating that ducks were utilizing glucuronate and could not generate ascorbate during caloric deficiency (Linster and Van Schaftingen, 2006). Glutaric acid was also found in higher concentrations during feed restriction. Glutaric acid is produced by amino acid metabolism and is correlated with metabolic acidosis during starvation (Wishart et al., 2018). Higher concentrations of glutaric acid in ducks experiencing a caloric deficit may be due to the utilization of alternative energy pathways.

During feed restriction, there was an increase in creatine, an amino acid that supplies energy to the cells, primarily the muscles (Hedemann and Damgaard, 2012). Muscle catabolism generally seen during severe malnutrition and occurs when there are no other sources of energy (Carbone et al., 2012). A study in rats found that 16-h fasting produced a 3-fold increase in serum creatine but did not measure muscle catabolism (Robertson et al., 2011). Ducks may have catabolized muscle tissue during feed restriction for energy, given the 8%–23% reduction in body mass.

## Metabolites associated with microbiome

While gut microbiota was not measured, metabolite changes suggest the possibility that microbes were altered during feed restriction. Starvation causes alterations in the mucosal structure and transport function of the small intestine, leading to changes in fluid and ion secretion (Ferraris and Carey, 2000). For example, a study in chickens found that a 70% reduction in feed caused changes in the gut microbiota composition (Yan et al., 2021). Additionally, a study in Asian seabass (*Lates calcarifer*) found that the intestinal microbial community composition changes in response to starvation in less than 3 days (Xia et al., 2014). The microbiome can achieve its previous microbial diversity after periods of starvation (Trinh et al., 2023). Metabolites associated with a healthy gut (arabinitol) were found in higher concentrations during the baseline. Arabinitol is a sugar alcohol produced specifically by *Candida spp*., a yeast-like fungus that is a ubiquitous inhabitant in healthy bird crops and gastrointestinal tracts (Talazadeh et al., 2022). Arabinitol is not absorbed efficiently by the intestine and is converted to pentose sugars by bacteria such as *Lachnospiraceae* and *Ruminococcaceae* (Ramirez et al., 2020). Decreases in arabinitol may indicate a change in the microbiome during food restriction.

During feed restriction, trehalose and trimethylamine (TMA) were higher; this suggests bacterial utilization of alternative energy sources and possible alteration of the gut microbiota. Trehalose is a sugar synthesized by numerous bacteria species, insects and plants. It is produced by bacteria such as *Escherichia coli, Corynebacterium sp*. and *Propionibacterium freudenreichii* (Strom and Kaasen, 1993; Cardoso et al., 2007; Ruhal et al., 2013). Trehalose has been found to accumulate in bacterial cells in response to stress. Trehalose can be metabolized from choline in the colon through host-microbial interaction (Cardoso et al., 2007; Rath et al., 2017). Alternatively, increases in trehalose may be associated with increased invertebrate consumption as trehalose is the primary blood sugar in insects (Becker et al., 1996). Although we found no relationship between the degree of mass loss and trehalose concentration, the ducks may have opportunistically consumed insects. Trimethylamine (TMA) has been shown to be produced by the gut microbiota from dietary quaternary amines such as choline and carnitine (Rath et al., 2017). Synthesis of TMA can be influenced by differences in gut microbiota (Delgado et al., 2006) and has been associated with severe cardiovascular disease in humans (Rath et al., 2017). It is possible that increases in trehalose and TMA may be associated with alterations in the gut microbiota in response to feed restriction in this study.

## Conclusion

This study demonstrated that mallards utilize endogenous reserves in times of limited caloric intake. We suggest that similar metabolism shifts would occur during migration and egg incubation in free-ranging mallards when energy use exceeds energy acquisition. As the ducks in this study were fed a commercial diet, extrapolating these results to wild mallards must be interpreted cautiously. Thus, more research is needed to investigate the response of the fecal metabolome to feed restriction in different life history stages of the annual cycle.

## Data Availability

The data presented in this study are openly available at Murray, Breanne (2022), “Utilizing NMR fecal metabolomics as a novel technique for detecting the physiological effects of food shortages, chronic stressors, in waterfowl”, Mendeley Data, V1, doi: 10.17632/yphnb98s2m.1.
